# The Role of Diet, Micronutrients and the Gut Microbiota in Age-Related Macular Degeneration: New Perspectives from the Gut–Retina Axis

**DOI:** 10.3390/nu10111677

**Published:** 2018-11-05

**Authors:** Emanuele Rinninella, Maria Cristina Mele, Nicolò Merendino, Marco Cintoni, Gaia Anselmi, Aldo Caporossi, Antonio Gasbarrini, Angelo Maria Minnella

**Affiliations:** 1 UOC di Nutrizione Clinica, Dipartimento di Scienze Gastroenterologiche, Endocrino-Metaboliche e Nefro-Urologiche, Fondazione Policlinico Universitario A. Gemelli IRCCS, Largo A. Gemelli 8, 00168 Rome, Italy; emanuele.rinninella@unicatt.it (E.R.); gaia.anselmi@gmail.com (G.A.); antonio.gasbarrini@unicatt.it (A.G.); 2 Istituto di Patologia Speciale Medica, Università Cattolica del Sacro Cuore, Largo F. Vito 1, 00168 Rome, Italy; 3 Laboratorio di Nutrizione Cellulare e Molecolare, Dipartimento di Scienze Ecologiche e Biologiche (DEB), Università della Tuscia, Largo dell’Università snc, 01100 Viterbo, Italy; merendin@unitus.it; 4 Scuola di Specializzazione in Scienza dell’Alimentazione, Università di Roma Tor Vergata, Via Montpellier 1, 00133 Rome, Italy; marco.cintoni@gmail.com; 5 UOC di Oculistica, Dipartimento di Scienze dell’Invecchiamento, Neurologiche, Ortopediche e della Testa-Collo, Fondazione Policlinico Universitario A. Gemelli IRCCS, Largo A. Gemelli 8, 00168 Rome, Italy; aldo.caporossi@unicatt.it (A.C.); angelomaria.minnella@unicatt.it (A.M.M.); 6 Istituto di Oftalmologia, Università Cattolica del Sacro Cuore, Largo F. Vito 1, 00168 Rome, Italy

**Keywords:** age-related macular degeneration, gut-retina axis, gut microbiota, dietary habits, micronutrients, fish oil, omega-3 polyunsaturated fatty acids, personalised medicine

## Abstract

Age-related macular degeneration (AMD) is a complex multifactorial disease and the primary cause of legal and irreversible blindness among individuals aged ≥65 years in developed countries. Globally, it affects 30–50 million individuals, with an estimated increase of approximately 200 million by 2020 and approximately 300 million by 2040. Currently, the neovascular form may be able to be treated with the use of anti-VEGF drugs, while no effective treatments are available for the dry form. Many studies, such as the randomized controlled trials (RCTs) Age-Related Eye Disease Study (AREDS) and AREDS 2, have shown a potential role of micronutrient supplementation in lowering the risk of progression of the early stages of AMD. Recently, low-grade inflammation, sustained by dysbiosis and a leaky gut, has been shown to contribute to the development of AMD. Given the ascertained influence of the gut microbiota in systemic low-grade inflammation and its potential modulation by macro- and micro-nutrients, a potential role of diet in AMD has been proposed. This review discusses the role of the gut microbiota in the development of AMD. Using PubMed, Web of Science and Scopus, we searched for recent scientific evidence discussing the impact of dietary habits (high-fat and high-glucose or -fructose diets), micronutrients (vitamins C, E, and D, zinc, beta-carotene, lutein and zeaxanthin) and omega-3 fatty acids on the modulation of the gut microbiota and their relationship with AMD risk and progression.

## 1. Introduction

Age-related macular degeneration (AMD) is a complex multifactorial disease, and in developed countries, it represents the first cause of legal and irreversible blindness among individuals aged ≥65 years. Globally, it affects 30–50 million individuals, and despite the introduction of new therapies for prevention and treatment, it is expected to increase by tenfold (300 million) by 2040 [1]. Its prevalence is relatively low in young-adult individuals; however, it reaches nearly 12% in individuals aged >80 years [2]. The disease affects the quality of life and daily living activities of the patients, and it causes human and social burdens, as well as high economic costs for the entire healthcare system [3,4].

AMD, in the early stages, is characterized by the presence of hyaline deposits, referred to as “drusen”, and hyper/hypopigmentations of the retinal epithelium in the retinal macular area, without visual impairment (age related maculopathy); however, it may evolve in the advanced dry form, referred to as “geographic atrophy” (GA), which is characterized by a significant loss of retinal pigment epithelium (RPE), or it may evolve in the wet (neovascular) form, sustained by abnormal choroidal neovascularization. These neovessels may undergo plasma or blood extravasation, leading to neurosensory or RPE detachment with fluid and/or blood accumulation. These changes, in turn, attract fibroblast migration and proliferation with further epithelium damage. At this stage, the GA or the neovascular form present moderate to severe visual loss.

It is unclear whether these forms are distinct or if they are different features of the same disease [5]. The neovascular form is treated with the use of anti-VEGF drugs; no effective treatments are available for the dry form, which relies on the prevention and control of risk factors, including specific nutritional intake and dietary supplements.

**Figure 1:**
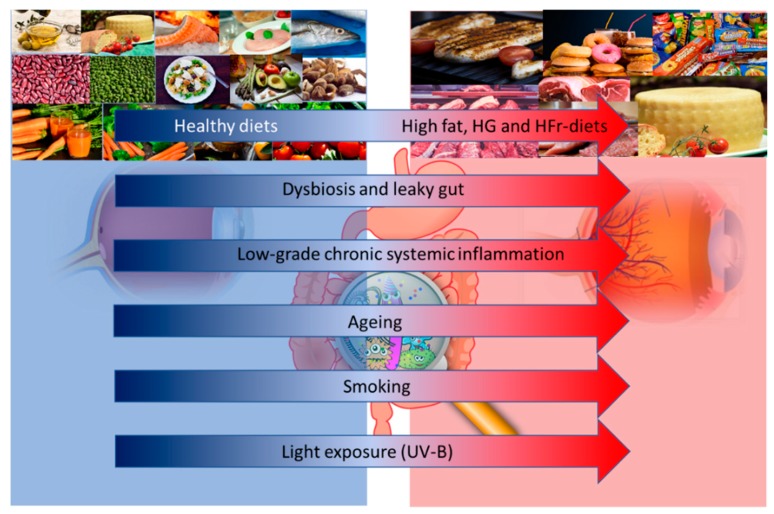
Altered dietary habits, dysbiosis and leaky gut, and low grade inflammation, together with aging, smoking and light exposure may influence the risk and progression of age-related macular degeneration (AMD). Abbreviations: HG: High Glucose; HFr: High Fructose; UV-B: Ultraviolet-B.

One potential approach to reduce the risk of AMD is the prescription of vitamins and other anti-oxidative micronutrients [6]. The main reason for this choice resides in their anti-inflammatory and anti-oxidant properties. Although the precise causes of AMD remain unknown, there is a clear role of inflammation in the pathophysiology of this disease. The RPE is fed by a dense vascular network, with high oxygen tension; moreover, a high rate of unsaturated fatty acid and photosensitizing compounds make the retina highly susceptible to reactive oxygen species (ROS) damage [7]. For example, one of the most recognized risk factors of AMD is light exposure: all light, even ambient natural light, can induce the formation of ROS in the RPE, which leads to the creation of lipid and protein peroxidation products [8]. Smoking is another well-known risk factor for AMD and is the most consistently identified modifiable risk factor, given its pro-oxidative and pro-inflammatory effect [5]. Many other factors, such as age, genetics, lifestyle, environmental factors and diet, may influence the risk and progression of AMD (Figure 1).

Obesity and overweight are associated with an increased risk of AMD in a dose-dependent fashion [9], while a reduction in the waist-to-hip ratio has been demonstrated to decrease the risk of AMD [10]. The impacts of high sugar and Western diets have also been shown in the risk and progression of AMD [11]. Furthermore, epidemiological studies strongly suggest the important contributions of several nutritional and non-nutritional compounds beyond the necessary energy intake to the risk of AMD. There has also been substantial progress in identifying the genetic variants that impact AMD risk.

Nutrients may act directly similar to antioxidant or anti-inflammatory compounds or indirectly by the gut microbiome; thus, there is a strong interest of the scientific community in the established efficacy of nutraceutics and functional foods rich in antioxidant and prebiotics in the prevention and as support to anti-AMD pharmacological treatments.

In the previous 10 years, an altered gut microbiota has been associated with many intestinal and extra-intestinal diseases, such as metabolic and inflammatory diseases, non-alcoholic fatty liver disease (NAFLD), cancer and obesity [12,13,14,15]. An increased intestinal permeability permits a higher translocation of bacterial products such as the endotoxin lipopolysaccharides (LPS) and pathogen-associated molecular pattern molecules (PAMPs), inducing low-grade inflammation in several tissues through the activation of pattern recognition receptors (PRRs). This biological crosstalk occurs also in dendritic cells, perivascular macrophages and RPE cells and may sustain ocular inflammation. Moreover, gut microbiota metabolites and products may modulate retina-specific immune cells [16]. Recently, an obesity-associated gut-microbiota has been shown to drive pathological angiogenesis toward aberrant choroidal neovascularization (CNV) in retinal tissue [16]. All this evidence supports the concept of a “gut–retina axis” in the pathogenesis of ocular diseases., It is known that the gut microbiota undergoes significant changes after the age of 65 years, reducing its richness and resilience to lifestyle changes, antibiotics, and diseases, particularly in frail patients [17] Also, the gut microbiota is a crucial player in the metabolism and absorption of several macro and micronutrients in the gut barrier, even those involved in AMD.

The identification of the relationship among diet, micronutrients, the gut microbiota, and host immunity is a new frontier in the treatment of many metabolic diseases. This review focuses on the interplay of the gut microbiota with diet and micronutrients in AMD, in the so-called “gut–retina axis”.

## 2. Literature Review Method

We searched and selected the most relevant publications between January 1998 and August 2018 on PubMed, Web of Science and Scopus with the search terms “age-related macular degeneration”, “gut microbiota”, “diet”, “micronutrients”, and “fish oil” and “omega-3 polyunsaturated fatty acids”. We selected all experimental and epidemiologic studies, supporting or not, the link between dietary habits, micronutrients, the gut microbiota, and AMD.

## 3. Diet and Gut-Microbiota in Age-Related Macular Degeneration (AMD)

### 3.1. Gut Microbiota: A Diet-Driven Ecosystem

The gut microbiota is a complex ecosystem colonizing the gastrointestinal tract with a higher distribution in the colon (10^14^ cells per gram of feces). The gut microbiota is composed of a large number of bacteria (up to 1000 different species), archaea, fungi, and viruses. Overall, the number of resident microbial cells is 1.3 times greater than the number of eukaryotic cells of the whole human organism [18]. The gut microbiota has continuous cross-talk with its host, modulating the process of food absorption and maintaining the physiologic stimulation of the gut immunity system.

Although there are many known bacterial phyla in the gut microbiota, only a few phyla are predominantly represented and account for more than 160 species: among them, Firmicutes (mainly Gram-positive), Bacteroidetes (mainly Gram-negative), Actinobacteria and Proteobacteria are prevalent [19]. The former two (Firmicutes and Bacteroidetes) account for more than 90% of all phylogenetic types in both mice and humans; the less represented phyla are Verrucomicrobia and Fusobacteria [15,20].

The gut microbiota composition may vary according to dietary habits. For example, *ob*/*ob* mice (mutant mice that eat excessively due to mutations in the leptin gene) have a 50% reduction in the abundance of Bacteroidetes and a proportional increase in Firmicutes compared with those in lean mice [21]. The provision of a high-caloric, high-fat and simple sugar-based diet (Western diet) to wild-type mice leads to an overall decrease in the diversity of the gut microbiota, with a specific reduction in Bacteroidetes and a bloom of a single class of Firmicutes (Mollicutes) [22]. Turnbaugh et al. showed, in a mouse model, that this “obesity-associated gut microbiome” had an increased capacity to harvest energy from the diet, breaking down otherwise indigestible dietary polysaccharides. Moreover, this property is a transmissible trait: adult germ-free mice colonized (by gavage) with a microbiota of obese (*ob*/*ob*) donors exhibited a significantly higher increase in body fat over two weeks than mice colonized with lean donors’ microbiota fed with the same quantity and caloric density of chow [15]. Moreover, a high-fat-diet (HFD) does not induce weight gain or hypercholesterolemia in the absence of gut microbiota, as shown in germ-free C57BL/6J mice compared to wild-type mice; germ-free mice also show enhanced insulin sensitivity with improved glucose tolerance compared to conventional mice on the same diet [23]. These findings confirm the pivotal role of the gut microbiota in driving the metabolic pathways of obesity and low-grade inflammation.

### 3.2. High-Fat Diets and the Gut Microbiota in AMD

It is well known that a HFD and unhealthy lifestyle may influence the progression of AMD in genetically predisposed individuals [24]. Chiu et al. specifically investigated the role of dietary patterns in the prevalence of AMD, collecting the nutritional habits of 4088 participants enrolled in the Age-Related Eye Disease Study (AREDS) (8103 eyes). The authors initially derived the dietary patterns by conducting principal component analysis (PCA) of food consumption data from the AREDS food frequency questionnaire (FFQ); they subsequently performed a logistic analysis and a qualitative comparative analysis (QCA) to evaluate the associations between dietary patterns and AMD, comparing the highest to lowest quintiles for each pattern. This cross-sectional study reported that a “Western diet”, mainly composed of high-fat dairy products, butter or margarine, gravies, processed and red meats, eggs, sweets and desserts, energy drinks, refined grains and French fries, was strongly associated with a higher prevalence of advanced AMD (OR: 3.70; 95% CI, 2.31–5.92; *p* < 0.0001); in contrast, the so-called “oriental” dietary habit, mainly composed of vegetables, legumes, rice, whole grains, fruit, tomatoes, green leafy vegetables, low-fat dairy, fish, and seafood, was protective from advanced AMD (OR: 0.38; 95% CI, 0.27–0.54; *p* < 0.0001) [25].

A large epidemiological study, conducted in 21,287 participants from the Melbourne Collaborative Cohort Study, confirmed abdominal obesity as an independent risk factor for early (OR: 1.13; 95% CI, 1.01–1.26; *p* = 0.03) and late AMD (OR: 1.75; 95% CI, 1.11–2.76; *p* = 0.02) [26].

Andriessen et al. released the first evidence of the critical role of the gut microbiota in exacerbating CNV from a HFD. The authors randomized six-week-old C57BL/6J mice on a regular chow diet (RD; 16% kcal fat) or a high-fat diet (HFD; 60% kcal fat). The two groups were further randomized to receive (or not) neomycin (a non-gut permeable antibiotic) in their drinking water at the ninth week. At the eleventh week, all mice were subjected to a laser photocoagulation that induces choroidal neovascularization, mimicking neovascular AMD. Following sacrifice, HFD-fed mice showed a 60% increase in CNV compared to RD-fed controls. Surprisingly, HFD-fed mice treated with neomycin displayed a CNV level similar to RD-fed control mice, even if weight gain was consistent with the other HFD-diet mice. In the authors’ opinion, this could explain the role of the gut microbiota in the aberrant choroidal neoangiogenesis, irrespective of the body weight.

Moreover, the type of diet significantly influenced the gut microbiota composition as shown by sequencing bacterial 16S rRNA extracted from the feces of mice: *Bacteroides* comprised 66% and Firmicutes comprised 33% of the total bacteria in RD-fed mice, whereas a sharp inversion of this ratio was found in HFD-fed mice (19% and 67%, respectively). Notably, neomycin-treated HFD-fed mice had a *Bacteroides* rate of 65% of the total bacteria (similar to RD-fed mice) and a Firmicutes rate of <10%. Of note, the HFD-diet harbored a modest presence of Actinobacteria and Spirochaetes. In turn, the different gut microbiota compositions in HFD-fed mice sustained an abnormal inflammatory response. Analysis via fluorescence-activated cell sorting (FACS) of retinas and sclera–choroid–RPE indicated a two-fold increase in mononuclear phagocytes and microglia of HFD-fed-mice compared to control RD-fed mice; in contrast, neomycin treatment abolished this effect. This gut dysbiosis-driven neovascular response was found to be linked to an increased intestinal permeability and chronic low-grade inflammation (elevated production of IL-6, IL-1beta, TNF-alpha and VEGF-A) [16]. It is not clear if the neomycin itself could act stabilizing Firmicutes rather than *Bacteroides* also in other animal contexts. However, the modulation of gut microbiota, even in HFD, appears a potential way to deal with AMD progression.

### 3.3. High-Glucose or -Fructose Diets and the Gut Microbiota in AMD

The excess of sugar in modern dietary habits has been linked to obesity and several metabolic diseases, including diabetes mellitus type II, NAFLD and cardiovascular diseases [27]. Inside these settings, a pivotal role is currently attributed to the gut microbiota, which is responsible for changes in intestinal permeability, leading to metabolic endotoxemia, insulin-resistance, lipid accumulation in tissues and organs and increasing plasmatic levels of inflammatory cytokines [28,29]. Moreover, many cross-sectional and prospective studies highlight a potential role of dietary carbohydrates in increasing the risk or progression of AMD, even if simple sugars play the most significant role in this context [30]. Their impact on the gut microbiota has recently been elucidated by Do et al. [31]: the authors assigned six-week-old C57BL/6J mice to receive four distinct dietary regimes: normal diet (ND), HFD, high-glucose diet (HGD), and high-fructose diet (HFrD). After 12 weeks, HFD- and HFrD-fed mice showed significantly higher plasmatic levels of blood glucose, total cholesterol, LDL and endotoxin than those of ND-fed mice. Moreover, HFD-, HGD- and HFrD-fed mice showed lower microbial diversity (fewer operational taxonomic units and lower Shannon indices) than ND-fed mice, with a lower abundance of Bacteroidetes and an increased abundance of Proteobacteria at the phylum level, as well as an increase of the *Desulfovibrio vulgaris* species. These metabolic and microbial differences were accompanied by a significant (2.5-fold greater) increase of gut permeability, demonstrated by a high plasma fluorescein isothiocyanate (FITC)-dextran concentration and a reduced expression of tight junction proteins, such as ZO-1 and Occludin. Consequently, the expression of inflammatory cytokines (TNF-alpha and IL-1beta) in the colon was higher in HFD-, HGD- and HFrD-fed mice than that in in ND-fed mice. This evidence suggests that a HGD and/or HFrD, as well as a HFD, can shape the gut microbiota, increasing the Firmicutes-to-Bacteroidetes ratio and the proportion of Proteobacteria, one of the best sources of lipopolysaccharides (LPS) [32]. Moreover, these dietary regimens significantly alter gut permeability, boosting metabolic endotoxemia and systemic inflammation through modulation of the gut microbiota. These changes were independent of body weight gain, which occurred in HFD, but not in HGD and HFrD.

A recent experimental study by Rowan et al. [33] explored the link among HGD, the gut microbiota and AMD in an animal model. To compare the effect on the RPE of the dietary glycemic load, the authors divided C57BL/6J wild-type mice to receive a high-glucose (HG) diet or low glucose (LG) diet for 12 months; half HG-fed mice were switched to the LG-diet after six months (HGxoLG mice). Histological features of dry AMD, such as photoreceptor cell damage, subretinal deposits, RPE vacuolation, hypopigmentation, thinning, and disorganization, were identified at the end of the study in the HG-fed diet but not in the LG-fed diet. Surprisingly, HGxoLG mice showed RPE features similar to LG-fed mice. Moreover, differences were noted in the gut microbial and metabolomic composition: LG-fed mice had similar amounts of Bacteroidetes and Firmicutes phyla, whereas HG-fed animals harbored bacteria of unknown classification.

Moreover, the microbiome of HG-fed mice was enriched in Firmicutes and Clostridia, which was related to a more advanced retinal damage score, whereas LG-fed mice showed an abundance of the Bacteroidales and Eysipelotrichi classes, which are both associated with protection against AMD features. Furthermore, the gut microbiota of HGxoLG-fed mice appears similar to that of LG-fed mice. The proof of an active role of the gut microbiota in AMD was also demonstrated by the assessment of microbial metabolites (serotonin, hippurate, tyrosine, and tryptophan), in which higher levels of serotonin were associated with protection against retinal damage in a diet dependent-manner. This study demonstrated a potential effect of an LG diet in reversing AMD features through modulation of the gut microbiota, paving the way for a novel nutritional approach to AMD.

## 4. Micronutrients

The crucial role of the gut microbiota in AMD is not only explained by the macronutrient quality, such as dietary habits; micronutrients also play an important role in shaping the gut microbiota, which, in turn, is an efficient player in mediating their protective effects. Table 1 reports the main human studies (case-control, cohort studies or randomized clinical trials) that have investigated the role of micronutrient intake in AMD.

Several nutritional supplements have recently been shown to reduce the risk of progression from early to late AMD [34]. The most important longitudinal studies to prove the effectiveness of vitamins and micronutrients in preventing the worsening of AMD remain the AREDS and AREDS2, which were both sponsored by the National Institutes of Health (NIH). The AREDS was an extensive, randomized, placebo-controlled, multicentric study, which enrolled 3640 patients aged 55–80 years with the diagnosis of AMD and followed the patients for a mean follow up of 6.3 years (1992–1998): the authors showed a 28% reduced risk (odds ratio (OR) = 0.72; 99% confidence interval (CI) = 0.52–0.98) of progression of AMD in patients who consumed a combination of high doses of vitamins C and E, beta-carotene and zinc [35]. In the second trial, AREDS2, published in May 2013, omega-3 fatty acids, as well as the antioxidants lutein and zeaxanthin (the major constituents of macular pigment, chemically similar to beta-carotene) were investigated for an additive effect in reducing the progression of AMD. The results did not show a further significant impact of these other micronutrients compared with the AREDS formulation alone. However, the AREDS2 design was quite intricate, and a real placebo group was not included. The authors concluded that lutein/zeaxanthin could be an appropriate beta-carotene substitute for safety reasons (an increased incidence of lung cancer was identified in former smokers in the beta-carotene group vs. no beta-carotene group), while the efficacy of omega-3 fatty acids remained unclear [36]. A secondary, exploratory subgroup analysis of the AREDS2 study performed a direct comparison of lutein/zeaxanthin vs. beta-carotene and showed a small yet significant risk reduction in the development of late AMD (hazard ratio (HR): 0.82; 95% CI: 0.69–0.96; *p* = 0.02) and neovascular AMD (HR: 0.78; 95% CI: 0.64–0.94; *p* = 0.01) in the lutein/zeaxanthin group. The authors concluded that lutein/zeaxanthin could be more appropriate than beta-carotene in the AREDS-type supplements [37].

### 4.1. Vitamins C and E

Vitamin C is a potent antioxidant and a cofactor for many enzymatic reactions. Moreover, vitamin E is considered the most effective scavenger of free radicals [58]. Vitamins C and E are non-enzymic antioxidants that protect against oxidative stress, a significant contributory factor in the progression of neovascular AMD [56]. Vitamin C is the most important water-soluble antioxidant in the human body and the primary antioxidant in the eye. Vitamin C is present at high concentrations in the cornea, central corneal epithelium, lachrymal film, vitreous humor and aqueous humor [59]. Vitamin E comprises a group of essential eight lipid-soluble compounds, including tocopherols and tocotrienols, capable of penetrating into cellular membranes through a hydrophobic side and donating a phenolic hydrogen to reduce free radicals, thus preventing the propagation of ROS and the peroxidation of cellular and subcellular membrane phospholipids. One of the primary therapeutic applications of vitamin E is NASH (non-alcoholic steatohepatitis), a hepatic inflammatory syndrome sustained by low-grade systemic inflammation and lipid peroxidation often associated with metabolic syndrome and insulin-resistance [60]. Alpha-tocopherol is the most studied form of Vitamin E and has been shown to reduce the biomarkers of total body oxidative stress and inflammation [61,62]. In contrast to other water-soluble vitamins (i.e., thiamin, biotin, or folate) that, although they cannot be synthesized de novo by our body, can be produced by the normal microflora of the large intestine, vitamin C cannot be synthesized de novo in humans; however, it is obtained from dietary sources (fruits and vegetables) via intestinal absorption with a Na+ dependent carrier-mediated process [63]. The main vegetal sources of vitamin E include wheat germ oil, extra virgin olive oil, hazelnuts, and peanuts; animal sources include fish, oysters, eggs, and butter.

An animal study on mice published in 1985 showed that ascorbic acid might play a role in protecting the retina from oxidative insults by light. The retinas of rats that received ascorbate supplement showed significantly lesser damage than the retinas of unsupplemented rats [64]. The idea that vitamins C and E could have a protective effect for age-related retinal disease results from several epidemiological and animal studies. In these studies, contradictory results are reported correlating the intake of vitamin E or C and the prevention or reduction of the risk of AMD.

A recent case-control study conducted by Braakhuis et al. indicated that a higher intake of vitamin C was associated with a reduced risk of oxidative stress-related eye diseases (OR: 0.63; 95% CI, 0.23–1.03; *p* = 0.022) [57].

Another case-control study that enrolled 161 neovascular AMD cases and 369 population-based control subjects demonstrated that dietary intake of alpha-tocopherol and vitamin C was associated with a reduced risk of neovascular AMD [56].

The Beaver Dam Eye study indicated a correlation between low vitamin E intake and developing large drusen [65], whereas the Eye Disease Case-control Study Group did not identify a significant interaction between the risk of AMD and vitamin E [38].

The reason for these contradictory results could be because four different tocopherols are available, each with different biological activities and absorptions.

The Eye Disease Control Study reported that lower Vitamin C plasma levels were related to an increased AMD risk. Moreover, high plasma concentrations were not protective, i.e., vitamin or total vitamin C consumption was not associated with a statistically significantly reduced risk of AMD, although a potentially lower risk of AMD was suggested [38]. Furthermore, the population cohort Blue Mountains Eye Study showed a positive correlation between vitamin E intake and AMD with an RR of incident late AMD for participants in the middle and highest tertiles of total vitamin E intake of 2.83 (95% CI, 1.28–6.23; *p* = 0.0099) and 2.55 (95% CI, 1.14–5.70; *p* = 0.022), respectively, compared with the lowest tertile.

Therefore, the link between neovascular AMD and the consumption of vitamin C and vitamin E (alpha -tocopherol) remains controversial, with some studies showing a significant relationship and other studies indicating no relationship.

The redox state strongly modulates the gut microbiota: the oxidative stress generated in a HFD mouse model was found to alter the gut microbiota composition, increasing *E. coli* and *Enterococcus* and decreasing *Lactobacilli* compared to those in the control group [66]. Altered expression of anti-inflammatory pathways, such as superoxide dismutase (SOD), has been reported in inflammatory bowel diseases (IBD) [67]. In IBD, there is an imbalance of the gut microbiota, with a decline in the diversity of Firmicutes (a specific decrease in the *Clostridium leptum* groups, particularly *Faecalibacterium prausnitzii*) and an increase of Proteobacteria (such as Enterobacteriaceae and specifically *E. coli*) [68]. Moreover, the relative abundance of Bacteroidetes is increased in Crohn’s Disease (CD) compared with healthy controls [69].

Previous studies have indicated that both vitamins C and E are protective following mucosal tissue damage in chemical-induced colitis models [70,71]. It has also been demonstrated that natural antioxidants may regulate the gut microbiota composition by scavenging excessive free radicals and supporting the cellular and humoral immune responses [72].

Recent findings in an animal model of ileal pouchitis suggest that an antioxidant diet, enriched in vitamins C and E, selenium, and retinoic acid, may reshape the gut microbial community toward an anti-inflammatory profile, mitigating mucosal inflammation. This capacity appears to be mediated by an increase in the relative percent of Bacteroidetes and a decrease in Firmicutes at the phylum level, with an overall increase in alpha-diversity (Shannon diversity index) [73]. This evidence is confirmed by a human study conducted in pregnant women in the second trimester, which showed that higher intakes of vitamin E were associated with a decrease in Proteobacteria and Firmicutes and an increase in Bacteroidetes [74]. The association between micronutrient intakes, including Vitamins C and E, and gut microbiota variations was furtherly assessed in a small group of free-living adults with stable cystic fibrosis (CF): the authors found that vitamin C and E intakes were positively correlated with Firmicutes and its lower taxa (i.e., Clostridium) and negatively associated with Bacteroidetes [75]. Clearly, the issue must be further elucidated, particularly at lower taxonomic levels; moreover, a recent study conducted on iron-deficient infants and toddlers showed an increase of the relative abundance of the genus *Roseburia* (phylum Firmicutes), a butyrate producer, in the group supplemented with iron + Vitamin E compared to that in the only iron-supplemented group [76].

A recent animal study conducted on early-weaned piglets explored the effects of an antioxidant blend, including vitamins C and E, on the oxidative stress generated by weaning stress. The study confirmed the antioxidant capacity of these micronutrients in scavenging free radicals and restoring the gut microbiota microenvironment, increasing *Lactobacillus* and *Bifidobacterium* counts, and decreasing *E. coli* counts in the gut environment [77]. However, the antioxidant property of these compounds appears to be empowered by their synergistic effect, according to the so-called “antioxidant network theory” [78].

### 4.2. Zinc

Zinc is a trace mineral responsible for the metabolism of nucleic acids, signal transduction, protein folding, and gene expression. Zinc is involved as a cofactor in more than 300 enzymatic reactions in vivo [79,80].

In the eye, zinc plays an important anti-oxidant role, and it is a cofactor of many active ocular enzymes, including superoxide dismutase and catalase. Zinc can be found at high concentrations in the human retina, RPE, and choroid; moreover, it is involved in the formation of electrical signals of photoreceptors. Low levels of zinc are associated with poor night vision and the degradation of RPE and photoreceptors [81].

Recent evidence indicates that zinc also has a close interaction with the complement system, which may further represent an important factor in determining the beneficial effects in AMD [82]. Moreover, observational studies have suggested that individuals who eat a diet rich in antioxidant minerals (selenium and zinc) may be less likely to develop AMD [83].

In vitro studies have suggested that AREDS vitamins and zinc supplementation attenuate angiogenesis and endothelial-macrophage interactions, thereby reducing the progression of AMD [84]. Finally, data from an animal model of light-induced retinal degeneration suggest that integration with zinc induces changes in gene expression, as well as enhances the antioxidative power in the retina and reduces the oxidative damage that arises from intense light exposure. [85].

A randomized, prospective trial [49] showed, after six months of treatment, an improvement of visual acuity and contrast sensitivity and a shortening of the macular light flash recovery times in patients who received zinc supplementation.

Several clinical studies have been designed to define the exact dose of zinc supplementation and its beneficial effects. Experimental data [86] suggest that low amounts of zinc protect RPE cells in culture from stress-induced effects, whereas higher amounts of zinc have the opposite influence. These effects are dependent, in part, on the “health status” of the cells. It also appears that zinc-induced death of RPE cells can be attenuated by compounds such as antioxidants (alpha-tocopherol, Trolox, and metipranolol) or cellular energy substrates (pyruvate and oxaloacetate). Therefore, a combination of zinc and antioxidants or energy substrates instead of zinc alone should represent a safer and more effective treatment for diseases, such as AMD.

AREDS indicated that 80 mg of zinc oxide, alone or in combination with antioxidants, significantly reduced the risk of progression to advanced AMD. AREDS2 showed that a low dose of zinc (25 mg) instead of a high dose (80 mg) displayed no difference in the primary outcome of progression to late AMD. The Blue Mountains Study, a population-cohort study, indicated a protective effect of dietary zinc intake for early or any AMD with a potential threshold effect (>15.8 mg/day) [48].

Zinc is absorbed mainly in the small intestine as well as in the stomach and large intestine via a non-specific, unsaturable diffusion-mediated mechanism and saturable carrier-mediated component [79]. Zinc is indispensable for the growth of most organisms. The amount of zinc inside cells is highly regulated, as too little zinc does not support growth, while too much zinc is toxic. Moreover, numerous bacterial cells require zinc uptake systems for growth and virulence [87]. Considering that the gut houses the majority of an individual’s microbes, in recent years, numerous animal studies have been conducted with the aim of elucidating the impact of dietary zinc on the gut microbiota. One study, which collected feces throughout a five-week time course of dietary zinc manipulation, demonstrated that excess nutritional zinc alters the diversity and structure of the gut microbiota in mice [88].

Another study, conducted on chickens, demonstrated that quantities of zinc in the gastrointestinal tract are reduced in conventional chicks compared to limited-flora chicks, which suggests that the microbiota affect the availability of this trace element [87].

A dramatic compositional and functional remodeling of the gut microbiota of *Gallus gallus* was assessed under chronic zinc deficient conditions [89]. Another study on pigeons [90] showed different effects of zinc-methionine supplementation on intestinal bacterial growth depending on the dosage: an increase of Bacillaceae, *Lactobacillus*, *Enterococcus* and *Bifidobacterium* populations and a decrease of *Escherichia coli* were identified at the dosage of 2 mg, whereas an overall lower count of *Lactobacillus*, *Enterococcus* and *Bifidobacterium* populations occurred at the dosage of 10 mg. Further studies confirm these data; Engberg et al. [91] showed that supplementation with zinc bacitracin significantly reduced the number of coliform bacteria. Ren et al. [92] identified a significant increase in the amounts of *Lactobacillus* and *Bifidobacterium* and a decrease in the amounts of *E. coli*, *Staphylococcus* and *Enterococcus* in the feces of dogs supplemented with zinc- enriched probiotics compared to those in controls.

### 4.3. Carotenoids

Carotenoids are pigments responsible for the yellow, orange, and red colors of many fruits and vegetables and are divided into two classes: xanthophylls (lutein, zeaxanthin and the isomer meso-zeaxanthin) and carotenes (alpha-carotene, beta-carotene, and lycopene). Apart from the carotenoids present in major foodstuffs (e.g., melon, carrots, eggs, shrimps, lobsters, and salmons), the human diet includes carotenoids from spices such as saffron, paprika, and annatto. Due to their intense orange to red colors, carotenoids are also widely used as colorants in the food-processing industry [93].

The bioavailability of carotenoids can be influenced by dietary and phytological factors, according to the different species of carotenoids, and depending on the molecular linkage, amount of carotenoids consumed in a meal, effectors of absorption and bioconversion, nutrient status of the host, genetic factors, host-related factors, and interactions [94].

The mechanism of carotenoid absorption starts with the mechanical and enzymatic disruption of the food matrix followed by their emulsification and micellization in the intestinal lumen. The mixed micelles are absorbed by the small intestinal epithelium (enterocytes) through simple diffusion. However, studies have suggested the existence of receptor-mediated transport of beta-carotene and lutein in the apical membrane of enterocytes [95,96,97].

#### 4.3.1. Beta-Carotene

Carotenoid supplementation can prevent and reduce the risk of AMD and other ocular diseases [98]. Carotenoids are potent antioxidants that able to reduce the systemic oxidative stress that influences the macula. Alpha-carotene, beta-carotene, and lycopene have been found in human RPE and choroid to protect these tissues against light-induced oxidative damage and locally produced free radicals.

Several studies have shown that carotenoids are widely used to treat oxidative stress-induced ocular diseases, such as AMD and cataract [40,99,100,101].

The retinal content of macular carotenoids is inversely associated with the incidence of AMD [102,103]. Moreover, clinical studies have shown that carotenoid supplementation can improve visual performance in some subjects [46,55,104].

The original AREDS formula containing beta-carotene has been demonstrated to reduce the progression to advanced AMD. However, when lutein/zeaxanthin replaced beta-carotene in the original AREDS formulation, an increase in efficacy was observed [105].

The initial enthusiasm for supplementation with beta-carotene was tempered by the emergence of evidence that high doses of beta-carotene (30 mg daily) could be harmful in smokers, causing an increase in the incidence of lung cancer. This side effect does not occur in non-smokers or when the carotenoid is administered in lower doses. Therefore, it is recommended to avoid supplementation with beta-carotene in smokers with AMD.

The question of whether beta-carotene has protective or harmful effects on AMD remains to be clarified. A recent case-control study indicated a protective association between dietary intake of beta-carotene and oxidative stress-related eye diseases (OR: 0.56; 95% CI, 0.15–0.98; *p* = 0.007) [57]. However, contradictory results originated from the Blue Mountains Study, which showed a significantly positive correlation between beta-carotene dietary intake and AMD (R: 1.36; 95% CI, 1.02–1.81 per 1-SD increase; *p* = 0.039), with a significant trend across increasing tertiles of dietary beta-carotene intake. [48].

Nevertheless, it remains clear that beta-carotene represents a protective agent against a variety of other chronic diseases or cardiovascular diseases.

The previously described study on patients affected by CF showed that high intakes of beta-carotene were negatively correlated with Bacteroidetes and positively correlated with Firmicutes and their lower taxa (e.g., *Clostridium*). It is plausible that antioxidant vitamin (such as beta-carotene) supplement may counteract the impact of increased oxidative stress on gut bacterial members [75].

A study conducted to identify functional alterations of the gut metagenome related to symptomatic atherosclerosis suggests that high levels of beta-carotene in the serum of healthy controls could be due to the potential production of this anti-oxidant by the gut microbiota [106]. Even if poor data are available on this topic, it is plausible to suggest that the anti-inflammatory effects of beta-carotene are also mediated by the gut microbiota or its transformation to Vitamin A.

#### 4.3.2. Lutein and Zeaxanthin

Lutein, zeaxanthin and the isomer meso-zeaxanthin are the predominant carotenoids and belong to the class of xanthophylls which accumulate in the retina and are constituents of the retinal macular pigment (MP) [107]. Lutein and zeaxanthin play a pivotal role in maintaining the morphology and function of the macula, displaying their antioxidant activity through the absorption of damaging blue light, neutralization of photosensitizers and active oxygen species, and scavenging of free radicals [108].

In the retina, meso-zeaxanthin can be found in the center, zeaxanthin in the mid-periphery and lutein in the periphery of the macula [109].

Lutein is a potent antioxidant: high dietary intakes enhance immune function and also reduce the risk of developing chronic diseases, such as cancer and cardiovascular diseases [110]. Moreover, studies have shown that a supplement containing lutein, zeaxanthin and blackcurrant extract has beneficial effects on visual functioning [39], and high MP provides protection against the development of many retinal diseases, particularly AMD; in contrast, low MP increases the risk of these diseases [53,111].

Most vegetables (spinach, kale, lettuce, asparagus, and broccoli) and pistachio nuts contain only lutein, whereas corn and eggs contain both lutein and zeaxanthin. Fat consumption (i.e., a salad dressing, extra virgin olive oil or whole eggs) together with carotenoid intake have been shown to increase the absorption and bioavailability of some carotenoids, such as lutein [112].

Xanthophylls (lutein, zeaxanthin, and meso-zeaxanthin) seem to be more easily released from the food matrix and more efficiently micellized than carotenes, such as beta-carotene, and then absorbed by intestinal cells. [113].

The roles of lutein and zeaxanthin have been investigated in cohort studies, case-control studies and clinical trials. Overall, evidence [44,47,52] suggests a significant association between lutein/zeaxanthin intake and risk reduction for advanced AMD, both neovascular AMD and geographic atrophy, while the correlation with early AMD is unclear.

Evidence also suggests that lutein/zeaxanthin intake may have a protective effect against AMD in patients with a high genetic predisposition [114]. The Carotenoids in Age-Related Eye Disease Study (CAREDS) trial indicates that specific genes related to xanthophylls are associated with the development of AMD: in particular, nine genes were found to be associated with AMD, seven of which were associated with either lutein/zeaxanthin levels in the serum and macula [115]. 

In the Rotterdam Study [51] and the Blue Mountains Eye Study [48], high dietary intakes of lutein/zeaxanthin reduced the risk of early AMD among participants at a high genetic risk; no similar association was identified for individuals with a low genetic risk.

The macular pigment optical density (MPOD) is a measure of the attenuation of blue light by the macular pigment; therefore, it is correlated with the amount of lutein/zeaxanthin in the macula.

An increased intake of lutein and zeaxanthin through diet or supplementation has been demonstrated to increase MPOD levels, improve visual function, and reduce the risk of age-related eye diseases [116].

An important clinical trial evaluating the impact of lutein on MPOD is the Lutein Antioxidant Supplementation Trial (LAST) [39]. The results of this study show that MPOD can be modulated in patients with AMD. Moreover, the visual acuity, visual function, photo-stress recovery time, and contrast sensitivity were significantly improved in the group that received 10 mg of lutein plus antioxidants compared to placebo or lutein alone. A meta-analysis published in 2016, which analyzed 20 randomized controlled trials, confirmed the significant benefits of lutein, zeaxanthin and meso-zeaxanthin supplementation on MPOD in AMD patients and healthy subjects with a dose-response relationship [117].

The Blue Mountains Study, based on a cohort of 3654 Australian patients, indicated that participants within the top tertile of dietary intake of lutein and zeaxanthin (≥942 g/day) compared with the remaining population were significantly less likely to develop neovascular AMD (RR: 0.35; 95% CI, 0.13–0.92; *p* = 0.033), and individuals above the median (743 g) were also less likely to develop indistinct soft or reticular drusen (RR: 0.66; 95% CI, 0.48–0.92; *p* = 0.013) [48].

The AREDS2 [36] study and its previously described secondary analysis [37] confirmed the role of lutein/zeaxanthin in reducing the risk of late AMD and neovascular AMD.

A dominant population of *Bifidobacteria* and *Lactobacilli* in the gut microbiota has been associated with many benefits on human health, such as the inhibition of gut pathogens [118], prevention of colon cancer [119], synthesis of vitamins and enhancement of the immune system [120].

In 2013, a phytotherapy study on humans [121] identified two products, composed of blackcurrant extract powder, lactoferrin, and lutein, as good prebiotics that significantly promoted the growth of *Bifidobacteria* and *Lactobacilli* and reduced other bacteria populations, such as *Bacteroides* spp and *Clostridium* spp. Moreover, these compounds were also demonstrated to decrease the activity of beta-glucuronidase, an enzyme involved in colorectal carcinogenesis [122].

### 4.4. Vitamin D

Vitamin D exists in two primary forms: vitamin D2 (ergocalciferol), derived from plants, and vitamin D_3_ (cholecalciferol), derived from animal sources (animal fats, eggs, and fish oil). An essential source of vitamin D_3_ in humans derives from skin exposure to sunlight (ultraviolet radiation, UV-B), by photolysis of 7-dehydrocholesterol. Once assumed or produced by the skin, vitamin D must be activated by two necessary hydroxylation processes in the liver (25(OH) hydroxylation) and kidneys (1,25(OH)_2_ hydroxylation).

Vitamin D is a secosteroid, which acts as a steroid hormone by binding to the vitamin D receptor (VDR) and activating the transcription of genes involved in mineral and bone homeostasis, cell proliferation, differentiation, and apoptosis. Another mechanism of action is the non-genomic pathway, which involves secondary messengers and cytosolic kinase systems in target cells expressing specific receptors on the cellular membrane. Vitamin D is well known for its role in bone mineralization via phosphorus and calcium homeostasis. However, the VDR is ubiquitously expressed in other body tissues, such as intestinal, immune, endothelial and smooth vascular cells [123,124]. Moreover, vitamin D has also been investigated for its role in the regulation of immune function, inflammation, control of cell proliferation and apoptosis; potential inverse associations between vitamin D and chronic diseases (cardiovascular, autoimmune and cancer diseases) have thus been suggested [125,126].

More recently, a role of vitamin D in AMD has been proposed: VDR and the renal hydroxylases involved in Vitamin D metabolism (CYP27B1 and CYP24B1) have been found in the retina, RPE, and choroid [127,128,129]. In these tissues, vitamin D may act as a paracrine/autocrine hormone, thereby regulating oxidation, inflammation and angiogenesis. A recent review by Layana et al. [130] exposed several plausible mechanisms of action of vitamin D on AMD pathophysiology: protection against oxidative stress due to the generation of free ROS and lysosomal enzymes; possible inhibition of amyloid beta protein deposits, considered a primary activator of the complement cascade and inflammation; suppression of pro-inflammatory cytokines secreted by macrophages and microglia; and inhibition of angiogenesis, mediated by the inhibition of the transcription of hypoxia-inducible factor (HIF-1), induction of endothelial cell apoptosis and inhibition of the production of metalloproteinase, MMP-9.

One potential role of vitamin D deficiency in AMD is supported by many observational, cross-sectional and case-control studies, which correlated serum vitamin D levels or dietary vitamin intake with the risk of early or late AMD, although the present studies are heterogeneous and prospective studies are currently lacking [130]. However, although there is no robust evidence of a causal role of vitamin D deficiency in AMD pathogenesis, a protective role of vitamin D against chronic retinal inflammation should be considered in dietary programs and future studies.

A pivotal role in low-grade systemic inflammation, a constant of all chronic inflammatory diseases, is exerted by the gut permeability. Prolonged inflammatory dietary habits and dysbiosis may lead to a leakage of the gut barrier, potential disruption of the blood–brain barrier, and neuroinflammation [131]. Vitamin D promotes a gut barrier function that protects the integrity of the intestinal barrier. Its actions range from the regulation of tight junction proteins to the suppression of gut epithelial cell apoptosis and the stimulation of the expression of antimicrobial peptides, such as defensins and cathelicidin, by epithelial cells and monocytes. Furthermore, it regulates gut immunity toward an anti-inflammatory pattern, inhibiting pro-inflammatory Th-1 and Th-17 cells and stimulating T regulatory cells in ulcerative colitis (UC). The VDR is highly expressed in the intestine, and a low VDR expression or dysfunction is frequently found in patients with IBD. Moreover, a vitamin D deficiency has been correlated with disease activity, inflammation and clinical relapse in IBD [132]. Vitamin D was recently found to regulate gut permeability and the gut microbiota composition from the embryonal age [133,134].

Recent human studies support the evidence of a gut microbiota modulation by Vitamin D. A cross-sectional study, which investigated the association between vitamin D intake and the gut microbiota composition in healthy subjects, found that individuals in the highest vitamin D intake group had more abundant *Prevotella* and less abundant *Haemophilus* and *Veillonella* species [135]. An interventional human study indicated that high dose vitamin D supplementation promotes a shift in the gut microbiota on the upper intestinal tract, increasing the bacterial richness and decreasing Gammaproteobacteria [136]. The effect of vitamin D intake on modulation of the gut microbiota is mainly found in inflammatory diseases, such as UC and CD, where its administration seems to reduce intestinal inflammation and increase the abundance of several beneficial bacterial strains [137,138]. In another model of inflammatory disease, CF, vitamin D-insufficient CF patients were found to harbor numerous, potentially pathogenic species compared with vitamin D-sufficient CF patients in the gut and airway microbiota. The same study randomized vitamin D-insufficient patients to receive vitamin D_3_ or placebo. After 12 weeks of treatment, the *Lactococcus* species were enriched in patients who received vitamin D_3_, whereas *Veillonella* and Erysipelotrichaceae were significantly enriched in patients treated with placebo [139]. Taken together, these preliminary results show a potential beneficial effect of vitamin D on the systemic inflammatory status through enforcement of the gut barrier and positive modulation of the gut microbiota. This evidence could potentially be found to support the AMD model given the recent findings of Andriessen et al. [16]. However, this hypothesis should be confirmed by further studies.

### 4.5. Omega-3 Fatty Acids

Several studies have shown that the supplementation of omega-3 polyunsaturated fatty acids (PUFAs) provides multiple health benefits against different chronic degenerative diseases, such as cardiovascular diseases [140], rheumatoid arthritis [141], IBD [142], depression [143] and cancer [144].

The omega-3 PUFAs eicosapentaenoic acid (EPA; C20:5 ω3) and docosahexaenoic acid (DHA; C22:6 ω3) are the two main bioactive forms in humans. These fatty acids can be synthesized from the dietary precursor, an essential fatty acid, linolenic acid (ALA, C18:3), even if the synthesis pathway is quite complex, and dietary uptake of EPA and DHA-rich foods (seafood sources, such as sardines or salmon, nuts and seeds) is recommended. However, EPA and DHA are widely used as nutritional supplements, often as nutraceuticals.

Aging is characterized by an increase in the concentration of some pro-inflammatory molecules in the circulation, a phenomenon that has been termed “inflammaging” [145,146]. Low-grade inflammation (LGI) is associated with the age-related decline of many functional systems and increased risks of ill-health, poor well-being and mortality.

Controlling LGI could prevent or reduce the age-related functional decline associated with mental health and wellbeing as well as retinal health. The human retina contains lipid profiles enriched in long-chain polyunsaturated fatty acids (LC-PUFAs) and very long-chain polyunsaturated fatty acids (VLC-PUFAs) that are essential for regular retinal structure and function [147].

Epidemiological, clinical and experimental studies show that dietary omega-3 PUFAs (DHA and EPA) are associated with a reduced incidence of AMD and have a protective role in AMD progression. A randomized prospective study, the Nutritional AMD Treatment-2 (NAT-2) trial, demonstrated that patients who achieved red blood cell membrane EPA/DHA levels were significantly protected against AMD compared with the placebo group, having low levels of EPA/DHA [43,54]. The Eye Disease Case-Control Study in the US demonstrated that a higher intake of omega-3 fatty acids is inversely associated with AMD (OR: 0.55; 95% CI, 0.32–0.95). Moreover, the reduction in the risk of AMD with a higher intake of omega-3 fatty acids was primarily identified among subjects with a linoleic acid intake (an omega-6 fatty acid) below the median (*p* < 0.001) [41]. The Blue Mountains Eye Study also demonstrated a protective effect of high fish consumption in reducing the risk of incidence of late AMD in individuals in the most top quintile of intake [42]. Other studies report a lower risk of developing central geographic atrophy and neovascular AMD in individuals who consume higher levels of EPA/DHA [45,50]. These findings are in contrast with those of the AREDS2 study, concluding that the addition of DHA + EPA to the original AREDS formulation (vitamin C, vitamin E, beta-carotene, zinc, and copper) did not further reduce the risk of progression of AMD. However, one potential explanation is that the design, setting intake or subjects of AREDS2 did not permit an adequate expression of the prophylactic potential of omega-3 PUFA.

Furthermore, many studies have demonstrated that increased levels of omega-3 fatty acids in the diet do not prevent or slow the progression of AMD in accordance with the AREDS 2 trial [148].

Summing up the current evidence, beneficial effects on macular physiology and protection against degeneration can be assumed considering the extraordinarily high concentration of long-chain-PUFAs in retinal cell membranes. In general, epidemiological studies support the recommendation that a dietary fatty fish intake (e.g., salmon, tuna sardine, mackerel, and trout) or fish oil supplements are associated with a lower risk of AMD; however, it is prudent to advise patients of the actual scientific uncertainty regarding their real effectiveness.

Different bacterial taxa modulate immune functionality toward a *pro* or *anti*-inflammatory pattern. Thus, the composition of the microbiota community determines, in part, the level of resistance to infection and susceptibility to inflammatory diseases. A reduction of healthy bacteria, such as *Lactobacilli* and *Bifidobacteria*, has been associated with many metabolic disorders, such as cardiovascular diseases, diabetes, and obesity, all associated with metabolic endotoxemia, due to lipopolysaccharides (LPS) translocation across the intestinal epithelium and systemic inflammation. Many of these conditions are elicited by a high-fat diet or Western dietary patterns, decreasing Bacteroidetes and increasing both Firmicutes and Proteobacteria [149].

High (saturated) fat diets also promote the blooming of endotoxin-producing and sulfate-reducing bacteria, such as *Bilophila wadsworthia* (Proteobacteria phylum), associated with mucus layer degradation, low-grade inflammation and insulin-resistance [150,151]. In contrast, a fish oil-based diet has been demonstrated, in a murine model, to promote *Lactobacillus* and *Akkermansia muciniphila* blooming, associated with reduced inflammation and gut barrier improvement [152]. Several human studies have shown that EPA- and DHA-enriched diets may restore the Firmicutes/Bacteroidetes ratio, boosting Lachospiraceae taxa, resulting in an increased SCFA synthesis [153,154]. The improvement of the gut intestinal barrier is also reported by a human study showing a direct correlation between a higher total intake of omega-3 PUFA and lower serum zonulin concentration, a protein of intestinal tight junctions, for which serum levels are the hallmark of chronic low-grade inflammation and leaky gut [155]. The impact of PUFA on the gut microbiota composition appears to be crucial from the time of gestation and early life; moreover, a reduction of omega-3 PUFA in the maternal diet has been associated with a significant depletion of Epsilonproteobacteria, Bacteroides, and *Akkermansia* and a higher relative abundance of *Clostridia* in offspring [156]. For further investigations on the impact of omega-3 PUFA on the gut microbiota, we refer the reader to a comprehensive review of the literature by Costantini et al. [157]. Recently, a model of transgenic mice that exhibited an endogenously high omega-3 PUFA tissue content and a lower omega-6 to omega-3 PUFA ratio (fat-1 mice) has been demonstrated to maintain a lean phenotype compared with the wild-type counterpart (WT-mice), even when fed a high fat/high sucrose diet. At the basis of these results, there is a lower gut permeability, as shown by the ZO-1 immunostaining (higher in transgenic mice) and the plasma LPS concentration (assessed by liquid chromatography-tandem mass spectrometry), which is lower in fat-1 mice that had low plasma LPS concentrations. The authors also identified several differences in the gut microbiota composition between the two models, with a significantly higher diversity level and a more abundant population of Verrucomicrobia phylum (*Akkermansia* genus) in fat-1 mice than in WT mice. More interestingly, WT mice colonized with fat-1 fecal microbiota exhibited a significant improvement in glucose tolerance, a lower total weight gain than their WT counterparts, and a reduction of intestinal permeability, comparable to fat-1 mice, after transplantation with fat-1 fecal microbiota [158]. This evidence highlights the role of -3 PUFA in modulation of the gut microbiota, thus opening new horizons in the treatment of metabolic and inflammatory diseases linked to leaky gut, such as inflammatory bowel disease, diabetes, obesity, cancer, neuropathologies and, likely, AMD.

## 5. Conclusions and Future Perspectives

AMD is an invalidating disease with an increasing incidence due to the higher prevalence of elderly individuals in Western countries. Currently, there are few treatments available to manage its course.

Many observational studies have shown the potential role of micronutrient supplementation in lowering the risk of progression of the early stages of AMD. Moreover, an animal model highlighted the role of high-fat and high simple-sugar diets on the development of AMD through a derangement of the gut microbiota that leads to systemic low-grade inflammation. Furthermore, recent evidence indicates a strict interaction between the gut microbiota and retina that is referred to as the “gut-retina axis”. A better understanding of the mechanisms that underlie this connection may help clinicians to prompt new models of personalized care of AMD based on the promotion of healthy nutritional habits and adequate micronutrient intake. These practices could modulate the gut microbiota toward a reduction of dysbiosis, leaky gut and LGI and, consequently, retinal damage. Further studies are required to elucidate whether modulation of the gut microbiota through dietary interventions can delay the course of this frequent disease in the clinical setting.

## Figures and Tables

**Table 1:** Main human studies investigating the role of micronutrients in AMD.

Year; Author [Ref.]	Study Design	Population	Micronutrients	Aims	Results
1994; Seddon J.M. et al. [38]	Case-control	356 subjects with advanced AMD and 520 control subjects	Vitamins A, C, E, Carotenoids	To assess association between dietary intake of carotenoids and vitamins A, C, and E and the risk of AMD.	Carotenoid intake reduces the risk of AMD (OR: 0.57; 95% CI, 0.35–0.92; *p* = 0.02); L and Z were most strongly associated with a reduced risk of AMD (*p* = 0.001). The intake of retinol was not appreciably related to AMD. Vitamin E or total vitamin C consumption or the intake of retinol were not associated with a reduced risk of AMD.
2004; Richer S. et al. (the LAST study) [39]	RCT	90 patients affected by atrophic AMD followed up in a period of 12 months: Group 1, L 10 mg; Group 2, L 10 mg + antioxidants/vitamins and minerals Group 3, placebo	L	To determine whether nutritional suppl. with L or L with antioxidants, vitamins, and minerals improves visual function in atrophic AMD.	Visual function and eye MPOD improved with L alone or L together with other nutrients. Patients who received the placebo had no significant changes in the measured findings.
2006; Delcourt C. et al. (the POLA Study) [40]	Cohort study	899 subjects ≥60 years, resident in Sète (Southern France)	L, Z	To assess association of plasma L/Z with the risk of AMD and cataract in the POLA Study.	The highest quintile of plasma Z was significantly associated with reduced risks of AMD (OR: 0.07; 95% CI, 0.01–0.58; *p* = 0.005), nuclear cataract (OR: 0.23; 95% CI, 0.08–0.68; *p* = 0.003) and any cataract (OR: 0.53; 95% CI, 0.31–0.89; *p* = 0.01). AMD was significantly associated with combined plasma L/Z (OR: 0.21; 95% CI, 0.05–0.79; *p* = 0.01) and tended to be associated with plasma L (OR: 0.31; 95% CI, 0.09–1.07; *p* = 0.04), whereas cataract showed no associations. Among other carotenoids, only beta-carotene showed a significant negative association with nuclear cataract, but not AMD.
2006; Seddon et al. (US Twin Study) [41]	Case-control	681 twins: 222 with AMD (intermediate or late stages) and 459 with no maculopathy	omega-3 PUFA	To evaluate modifiable risk and protective factors for AMD among elderly twins.	Dietary omega-3 PUFA intake inversely associated with AMD (OR: 0.55; 95% CI, 0.32–0.95). Cigarette smoking increases risk, while fish consumption and omega-3 fatty acid intake reduce risk of AMD.
2006; Chua B. et al. [42]	Cohort study	2335 subjects ≥49 years, participated in the Blue Mountains Eye Study (1992–1994) and re-examined after 5 years (1997–1999)	omega-3 PUFA	To assess association between dietary fat intake in the older cohort and incident AMD.	Participants with omega-3 PUFA intake had a lower risk of incident early AMD (OR: 0.41; 95% CI, 0.22–0.75). A 40% reduction of incident early AMD was associated with fish consumption at least once per week (OR: 0.58; 95% CI, 0.37–0.90), whereas fish consumption at least 3 times per week could reduce the incidence of late AMD (OR: 0.25; 95% CI, 0.06–1.00).
2007; SanGiovanni et al. (AREDS Study Report 20) [43]	Case-control	4519 AREDS Study participants: 4 AMD severity groups and a control group	omega-3 PUFA	To assess association of lipid intake with AMD in AREDS Study.	Dietary total omega-3 PUFA intake was inversely associated with neovascular AMD (OR, 0.61; 95% CI, 0.41–0.90). Higher intake of omega-3 PUFAs and fish was associated with decreased likelihood of having neovascular AMD.
2007; SanGiovanni et al. (AREDS Study Report 22 [44]	Case-control	4519 AREDS participants: 4 AMD severity groups and a control group	L/Z Vitamins A and C, alpha-tocopherol	To evaluate the relationship among dietary carotenoids, vitamins A and C and alpha-tocopherol with prevalent AMD.	Dietary L/Z intake was inversely associated with neovascular AMD (OR: 0.65; 95% CI, 0.45–0.93), geographic atrophy (OR: 0.45; 95% CI, 0.24–0.86), and large or extensive intermediate drusen (OR: 0.7; 95% CI, 0.56–0.96), comparing the highest vs lowest quintiles of intake.
2008; SanGiovanni et al. (AREDS Study Report 23) [45]	Cohort study	2132 subjects from the clinical trial AREDS	omega-3 PUFA	To examine the association of neovascular AMD and CGA.	Decreased risk of progression from bilateral drusen to CGA among individuals who reported the highest levels of EPA (OR: 0.44, 95% CI, 0.23–0.87) and EPA+DHA (OR: 0.45, 95% CI, 0.23–0.90) consumption.
2008; Stringham, J.M. et al. [46]	Cohort study	40 healthy subjects	L, Z	To measure MPOD after L and Z supplementation for 6 months and evaluate visual improvement.	After 6 months, daily L/Z supplementation significantly increased MPOD and improved visual performance in glare for most subjects. At the 2-month time point, average MPOD had increased from 0.41 at baseline to 0.46. MPOD continued to increase at 4-month (*p* = 0.032) and 6-month (*p* = 0.003) time points, with increases from baseline of 0.10 and 0.16, respectively.
2008; Cho E. et al. [47]	Cohort study	71494 women and 41564 men with no diagnosis of AMD or cancer	L/Z	To assess association between L/Z intake and AMD risk by smoking status, vitamin C and E intakes, and body fatness.	L and Z intake was not associated with the risk of self-reported early AMD. There was a non-significant association between lutein and zeaxanthin intake and neovascular AMD risk.
2008; Tan J.S. et al. (the Blue Mountains Eye Study) [48]	Cohort study	3654 participants	Carotenoids, Vitamins A, C, E, Iron, Zinc	To assess incidence of early, late, and any AMD.	L/Z: participants in the top tertile of intake had a reduced risk of incident neovascular AMD (RR: 0.35; 95% CI, 0.13–0.92), and individuals with above median intakes had a reduced risk of indistinct soft or reticular drusen (RR: 0.66; 95% CI, 0.48–0.92). Beta-carotene: the highest compared with the lowest tertile of total beta-carotene intake predicted incident neovascular AMD (RR, 2.68; 95% CI, 1.03–6.96). Zinc: the RR that compared the top decile intake with the remaining population was 0.56 (95% CI, 0.32–0.97) for any AMD and 0.54 (95% CI, 0.30–0.97) for early AMD. Vitamin E: higher intakes predicted late AMD (RR compared with the lowest tertile, 2.83; 95% CI, 1.28–6.23; and RR, 2.55; 95% CI, 1.14–5.70 for the middle and highest tertiles, respectively).
2008; Newsome D.A. [49]	RCT	40 subjects randomly assigned to ZMC (25 mg) or placebo	Zinc (ZMC)	To assess association between use of ZMC and macular function in individuals with dry AMD.	After 6 months, the ZMC group showed improved visual acuity (*p* < 0.0001). ZMC (25 mg) twice daily was well tolerated and improved with macular function AMD.
2009; San Giovanni et al. (AREDS Study Report 30) [50]	Cohort study	1837 AREDS participants	omega-3 PUFA	To assess the association of dietary omega-3 PUFAs with progression to advanced AMD in subjects with a moderate risk of developing AMD.	Participants who reported highest omega-3 PUFA intake were 30% less likely than their peers to develop central geographic atrophy and neovascular AMD. Respective multivariate ORs are 0.65 (95% CI, 0.45–0.92; *p* = 0.02) and 0.68 (95% CI, 0.49–0.94; *p* = 0.02).
2011; Ho L. et al. (the Rotterdam Study) [51]	Case control	2167 individuals from the population-based Rotterdam Study	Zinc, beta-carotene, L/Z, EPA/DHA	To investigate the role of dietary nutrients in reducing the genetic risk of AMD conferred by the genetic variants CFH Y402H and LOC387715 A69S.	High dietary intake of nutrients with antioxidant properties reduces the risk of early AMD in individuals at high genetic risk. The results supported the possibility of biological interactions among LOC387715 A69S and CFH Y402H and zinc, beta-carotene, lutein/zeaxanthin, and EPA/DHA (all *p* < 0.05).
2012; Snellen E.L.M. et al. [52]	Case-control	72 cases and 66 controls	L	To assess the association between low antioxidant intake and neovascular AMD.	The prevalence rate of AMD in patients with low antioxidant intake and low L intake was approximately twice as high as that in patients with high intake: OR: 1.7; 95% CI, 0.8–3.7 and OR: 2.4; 95% CI, 1.1–5.1, respectively.
2012; Nolan J.M. et al. [53]	Cohort study	828 healthy subjects	L/Z	To investigate MPOD with respect to risk factors for AMD.	A statistically significant age-related reduction in MPOD was present in current and past smokers (*p* < 0.01), with a family history of AMD (*p* < 0.01). The enhanced risk that these variables represent for AMD may be attributable, at least in part, to a parallel deficiency of macular carotenoids.
2013; Souied E.H. et al. [54]	RCT	263 patients with early lesions of AMD received 840 mg/day of DHA and 270 mg/day of EPA or placebo	EPA, DHA	To evaluate the efficacy of DHA-enriched oral supplementation in preventing exudative AMD (time to occurrence of CNV, incidence of CNV developing in patients, changes in visual acuity, occurrence and progression of drusen, and changes in EPA plus DHA level in red blood cell membrane in RBCM).	Time to occurrence and incidence of CNV in the study eye were not significantly different between the DHA group (19.5 ± 10.9 months and 28.4%, respectively) and the placebo group (18.7 ± 10.6 months and 25.6%, respectively). In the DHA group, EPA plus DHA levels significantly increased in RBCM (+ 70%; *p* < 0.001). In the DHA- allocated group, patients steadily achieving the highest tertile of EPA plus DHA levels in RBCM had a significantly lower risk (−68%; *p* = 0.047; HR: 0.32; 95% CI, 0.10–0.99) of CNV developing over 3 years. No marked changes from baseline in best-corrected visual acuity, drusen progression, or geographic atrophy in the study eye were observed throughout the study in either group.
2014; Hammond, B.R. et al. [55]	RCT	115 young, healthy subjects (58 assigned to placebo and 57 assigned to L/Z (10/2 mg/day)	L, Z	To assess correlation between L and Z supplementation with MPOD, glare disability, photostress recovery, and chromatic contrast.	MPOD significantly increased in L/Z group versus placebo (*p* < 0.001 for both L and Z). Serum L and Z significantly increased by the first follow-up visit (at 3 months) and remained elevated throughout the intervention period of 1 year. There was a significant correlation between MPOD levels over time and visual performance.
2016; Aoki A. et al. [56]	Case -control	161 neovascular AMD cases and 369 control subjects	omega-3 PUFA, -tocopherol, Zinc, Vitamins D and C, beta-carotene	To assess association between micronutrient intake and neovascular AMD.	Low intakes of omega-3 PUFA, alpha-tocopherol, zinc, vitamin D, vitamin C, and beta-carotene were associated with neovascular AMD (*p* < 0.0001 for *n*-3 fatty acid, *p* < 0.0001 for alpha-tocopherol, *p* < 0.0001 for zinc, *p* = 0.002 for vitamin D, *p* = 0.04 for vitamin C, and *p* = 0.0004 for beta-carotene).
2017; Braakhuis A. et al. [57]	Case-control	149 controls; 42 cases with oxidative stress-related AMD	beta-carotene, Vitamin C	To assess association between the intake of dietary antioxidants and incidence of AMD.	Protective associations with higher consumption of vitamin C (OR: 0.63; 95% CI, 0.23–1.03; *p* = 0.022) and beta-carotene (OR: 0.56; 95% CI, 0.15–0.98; *p* = 0.007).

Abbreviations: AMD: age-related macular disease; CGA: central geographic atrophy; CNV: choroidal neovascularization; DHA: docosahexaenoic acid; EPA: eicosapentaenoic; L: Lutein; LAST study: Lutein Antioxidant Supplementation Trial study; MP: macular pigment; MPOD: macular pigment optical density; POLA study: Pathologies Oculaires Liées à l’Age study; RBCM: red blood cell membrane; RCT: randomized controlled trial; Z: Zeaxanthin; ZMC: zinc monocysteine; omega-3 PUFA: omega-3 polyunsaturated fatty acids.
